# Moisture-induced power generator fabricated on a lateral field-excited quartz resonator

**DOI:** 10.1038/s41598-024-61669-0

**Published:** 2024-05-11

**Authors:** Hyerim Baek, Jihun Choi, Sangmin Jeon

**Affiliations:** Department of Chemical Engineering, Pohang University of Science and Technology (POSTECH), 77 Cheongam-Ro, Pohang, Gyeongbuk Republic of Korea

**Keywords:** Energy science and technology, Engineering, Nanoscience and technology

## Abstract

We fabricated a moisture-induced power generator on a lateral field-excited quartz resonator to simultaneously measure changes in mass and voltage generation during water vapor adsorption. Circularly interdigitated gold electrodes were vacuum deposited on the top surface and used to measure changes in mass, and two symmetric semicircular gold electrodes were vacuum deposited on the bottom surface and used to measure changes in voltage generation. After coating a thin film of a mixture comprising sodium alginate, carbon black, and polyvinyl alcohol (SCP) on the top surface, an electric field was applied to create a concentration gradient of sodium ions between the interdigitated electrodes. The changes in the resonant frequency and voltage generation of the SCP-coated quartz resonator were measured simultaneously under various relative humidity conditions. The results revealed, for the first time, three distinct voltage-generation regions during moisture adsorption: (i) a region of negligible voltage generation, (ii) that of an increase in voltage generation, and (iii) that of a decrease in voltage generation.

## Introduction

Moisture-induced power generators (MPGs) have emerged as promising and sustainable energy-harvesting devices that use the ubiquitous environmental moisture to generate electricity. MPGs produce a direct current (DC) output that is suitable for conventional electronic devices. The operation of MPG begins with the adsorption of water molecules from the atmosphere, leading to the dissociation of charged ions from functional groups and their subsequent migration along the ion concentration gradient^1–3^ Although monitoring the actual amount of water adsorption into an MPG is crucial for understanding voltage generation, most studies have focused on the water uptake capabilities of materials under equilibrium relative humidity (RH) conditions^4,5^ or on the comparison of water adsorption rates of different materials^6^. Since water adsorption into a solid is neither instantaneous nor linear, it is critical to develop a method for simultaneously measuring real-time changes in water adsorption and voltage generation.

A quartz crystal microbalance (QCM) is a highly sensitive gravimetric sensor capable of measuring real-time mass changes with nanogram resolution (e.g., 17.7 ng·cm^−2^·Hz^−1^ for a 5 MHz crystal). Integration of an MPG with a QCM allows the monitoring of real-time changes in voltage generation during moisture adsorption. Liu et al. investigated the performance of a protein nanowire-based MPG using a nanowire-coated QCM to measure moisture adsorption^7^. However, the conventional QCMs, with circular gold electrodes that are positioned on both sides of the crystal, posed challenges in integration with other electrical devices. Unlike the conventional QCMs, a lateral field-excited (LFE) quartz resonator has two symmetric semicircular gold electrodes only on the bottom surface. Thus, independent MPG electrodes can be fabricated on the top surface of the resonator. This arrangement enables the simultaneous measurements of changes in adsorbed mass and corresponding electrical properties without any time delay, which allows for understanding the direct and quantitative effect of moisture adsorption on voltage generation. In previous studies, we fabricated interdigitated electrodes (IDEs) on an LFE quartz resonator and used the resonator as a gas sensor to concurrently measure changes in electrical resistance and mass during gas adsorption^8,9^. However, the results demonstrated only the potential of the device as a simple electrical resistance sensor, and IDE and LFE had different active areas.

In this study, we examined the real-time influence of water adsorption on the voltage generation performance of MPG by employing a circular IDE (CIDE)-patterned LFE quartz resonator, for the first time to the best of our knowledge. After coating a thin film of a mixture comprising sodium alginate (SA), carbon black (CB), and polyvinyl alcohol (PVA) on the CIDE-patterned surface, a concentration gradient of sodium ions was created between the IDEs by applying an electric field. The real-time measurement of simultaneous changes in the voltage output and resonant frequency revealed three distinct voltage-generation regions during moisture adsorption.

## Methods

### Materials

Alginic acid sodium salt from brown algae (MW 80,000–120,000), polyvinyl alcohol (MW 25,000, 88% hydrolyzed), and gold etchant were purchased from Aldrich (St. Louis, MO). Ammonium cerium (VI) nitrate and nitric acid were purchased from Samchun and Jusei, respectively. Deionized (DI) water (18.3 MΩ cm) was obtained using a reverse osmosis water system (Human Science, Korea). The 5 MHz quartz crystals (QCs) with a diameter of 1.27 cm were purchased from ICM (Oklahoma City, OK). Prior to use, the existing gold electrode and chromium layer were removed using the gold etchant, ceric ammonium nitrate, and nitric acid solution, respectively.

### Deposition of gold electrodes on QCs

**Figure 1:**
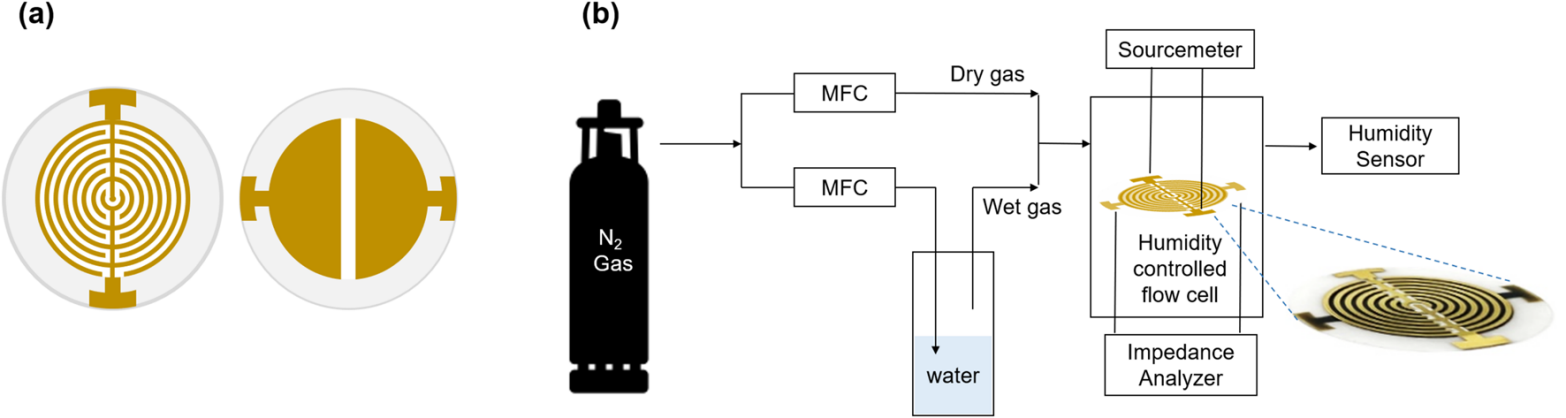
Schematic illustrations of the electrode patterns on (**a**) the top surface (IDE), (**b**) the bottom surface of a quartz resonator, and (**c**) the experimental setup. The inset photo shows an image of an IDE-coated lateral field-excited (LFE) resonator.

The shadow metal masks used for patterning the CIDEs and LFE electrodes were purchased from MPlex (Gyeonggi-do, Korea). The gold electrodes (Fig. 1a), were deposited onto both the top and bottom surfaces of the bare QC using a vacuum evaporator. LFE electrodes, which were intended for mass measurements and deposited on the bottom surface of the bare QC, had a diameter of 10 mm and a gap distance of 1 mm between the two semicircular electrodes. Circular CIDEs were deposited on the top surface to match the active area of the LFE electrode. The outer diameter, electrode width, and gap distance between the neighboring electrodes of CIDE were 10 mm, 300 µm, and 300 µm, respectively.

### Preparation of the SCP film on the CIDE-patterned LFE QC

The SCP film is composed of sodium alginate (SA), carbon black (CB), and polyvinyl alcohol (PVA). CB with high electrical conductivity was synthesized via flame pyrolysis using ghee butter as the precursor^10^. CB (10 mg) was added to 1 mL of a 2 wt.% PVA aqueous solution and then subjected to ultrasonication to obtain a homogenized solution. Subsequently, the solution was supplemented with 1 mL of a 4 wt.% SA aqueous solution, followed by additional ultrasonication to ensure thorough mixing. The resulting solution was applied to a CIDE-patterned LFE QC and spin-coated at 3,500 rpm for 30 s to form a thin film of thickness 180 nm on the QC (i.e., SCP–QC). The SCP–QC was subjected to ion polarization to establish an ion concentration gradient across the CIDEs, thereby enabling the SCP film to function as an MPG. During the ion polarization process, a voltage of 1 V was applied to the CIDE for 300 s after exposing the SCP–QC to 90% RH.

### Instrument setup for measuring real-time changes in moisture adsorption and voltage outputs.

Figure 1b shows a schematic of the instrument setup used in this study. The SCP–QC was placed inside a humidity-controlled flow cell that was connected to inlet and outlet tubes to realize a controlled gas flow. Dry nitrogen was used as the carrier gas, and the RH was regulated by adjusting the ratio of amount of dry nitrogen and wet nitrogen passing through a gas bubbler containing DI water. The total flow rate was fixed at 120 mL/min. The RH was measured using a humidity sensor (TSP01, Thorlabs, New Jersey) that was connected to the outlet tube. The two ends of the CIDEs located on the upper surface of the QC were connected to a SourceMeter (2636B, Keithley Instruments, Cleveland) for the continuous measurement of open-circuit voltage over time. Concurrently, the two ends of the LFE electrodes located on the bottom surface of the QC were connected to an impedance analyzer (QCM Z500, KSV Instruments, Finland) to measure changes in the resonant frequency.

## Results and discussion

**Figure 2:**
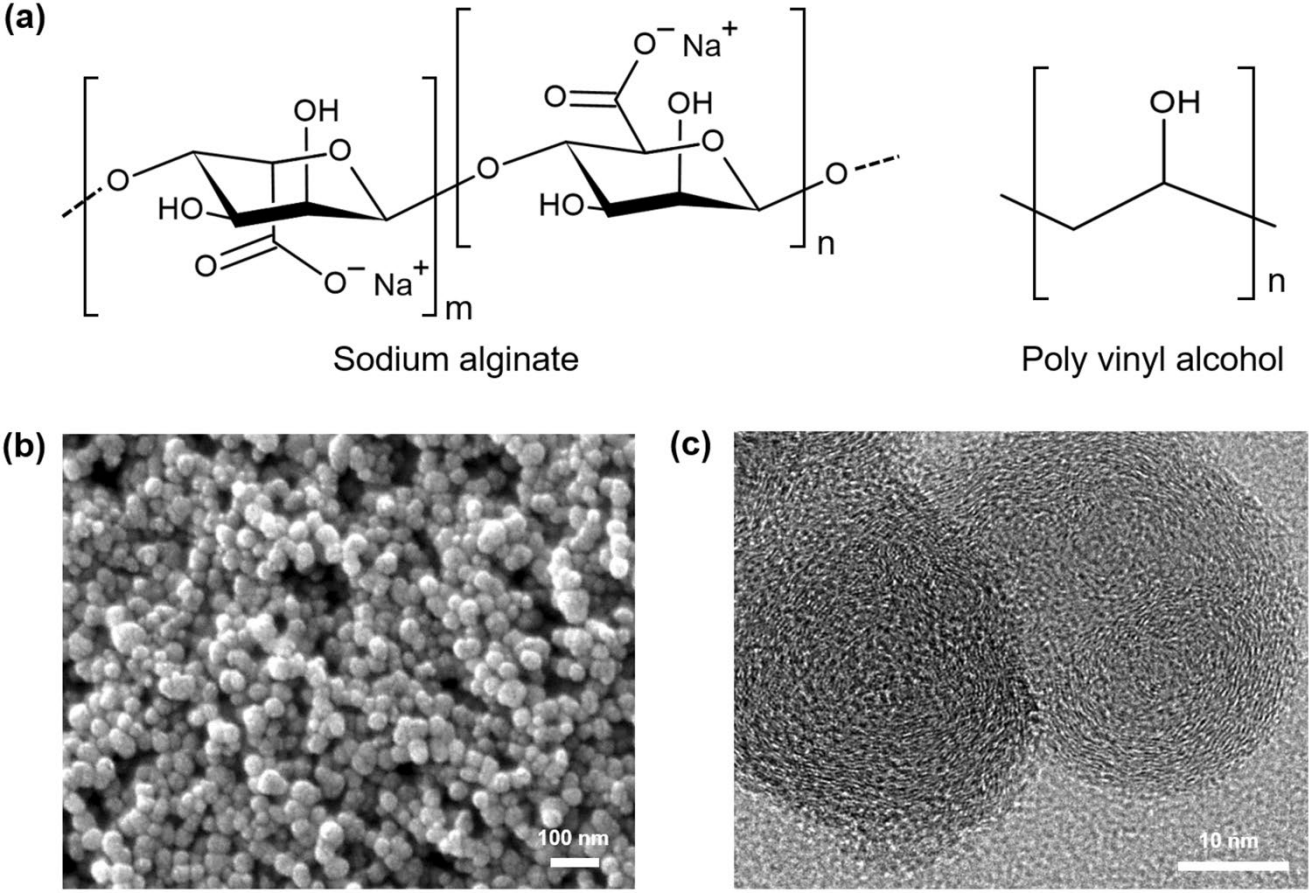
(**a**) Molecular structures of sodium alginate (SA) and polyvinyl alcohol (PVA). (**b**) SEM and (**c**) TEM images of the as-synthesized carbon black (CB) nanoparticles.

Figure 2a shows the molecular structures of SA and PVA, which are constituents of the SCP film. SA, which contains hydroxyl groups and sodium carboxylate groups (–COONa) in the polysaccharide backbone, contributes to the hydrophilicity of SCP and generates positively charged mobile sodium ions. Upon exposure to moisture, sodium ions undergo hydration and dissociation from the carboxylate groups. The application of an electric field between the electrodes results in a sodium ion concentration gradient between the electrodes. PVA, which is adhesive and contains hydroxyl groups (–OH) on every alternate carbon atom along the polymer backbone, improves the film-forming ability and the hydrophilicity of SCP. Figures 2b,c show the scanning electron microscopy (SEM) and transmission electron microscopy (TEM) images of the CB synthesized via flame pyrolysis. CB is spherical with a diameter of approximately 40 nm (Fig. 2b). Each particle has a primarily carbon-based structure with relatively ordered graphitic layers surrounding an amorphous carbon core (Fig. 2c). Because of this structure, the synthesized CB is electrically conductive, facilitating the transmission of the electric field into the SCP film between electrodes and contributing to the efficient polarization of sodium ions. Furthermore, the negative zeta potential (− 40.2 mV) of the CB surface (Supplementary Fig. S2) plays a crucial role in facilitating the polarization of sodium ions by offering binding sites for dissociated sodium ions.

**Figure 3:**
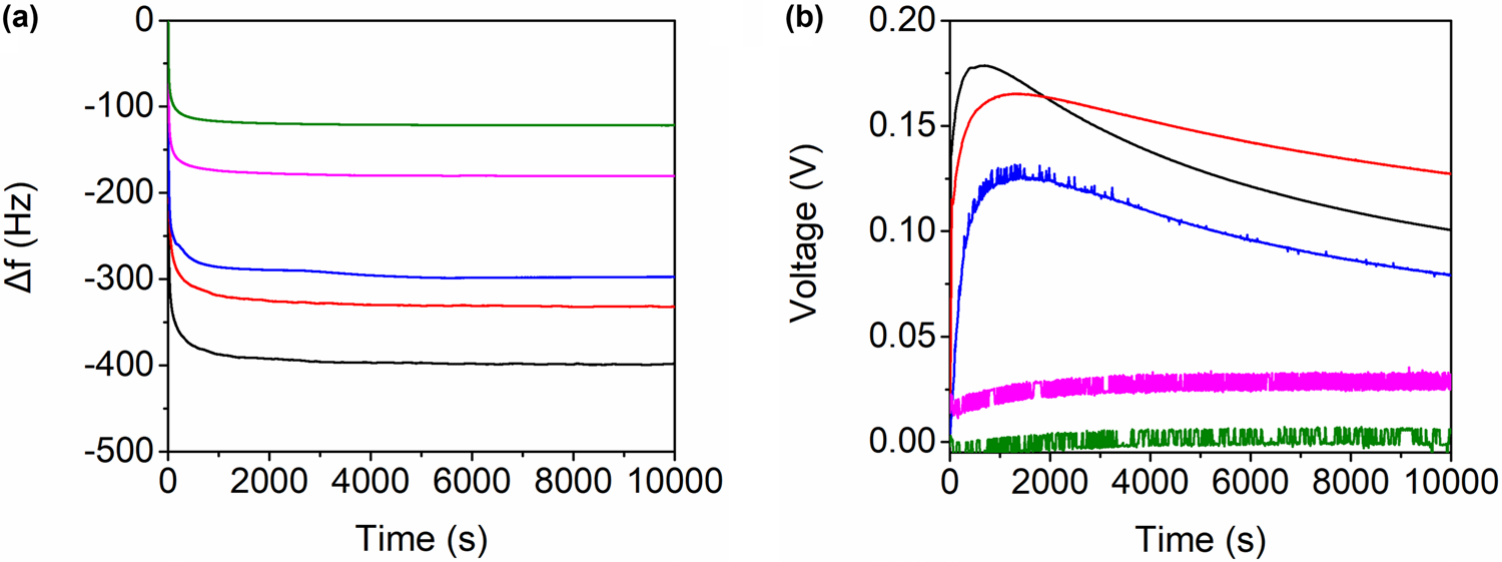
Time-dependent changes in (a) resonant frequency and (b) open-circuit voltage of the SCP–QC exposed to different RH values: 80% (black), 70% (red), 60% (blue), 50% (pink), and 40% (green).

Figure 3a shows the time-dependent changes in the resonant frequency under various RH conditions. The resonant frequency decreased as humidification was initiated, and a higher RH led to a more pronounced decrease. For a thin and rigid film, the change in the resonant frequency of a QC oscillating in the thickness-shear mode is directly related to the mass change according to the Sauerbrey Eq. ^11^:

$$\Delta f=-\frac{2{{f}_{0}}^{2}}{A\sqrt{{\rho }_{q}{\mu }_{q}}}\Delta m$$

where *f*_0_ is the resonant frequency of the unloaded quartz crystal; *ρ*_q_ is the density of quartz; *μ*_q_ is the shear modulus of quartz; and *A* is the active area of the electrode. Figure 3b shows the time-dependent changes in the voltage output under various RH conditions. The voltage output was negligible at 40% RH and increased to only 0.03 V as RH increased to 50%. However, there was a substantial increase in the voltage output as RH increased from 50 to 60%. At higher RH values (≥ 60%), the voltage output increased rapidly upon the initiation of humidification, reached its maximum value, and decreased subsequently. The maximum voltage values were 0.13 V at 60%, 0.16 V at 70%, and 0.18 V at 80%.

**Figure 4:**
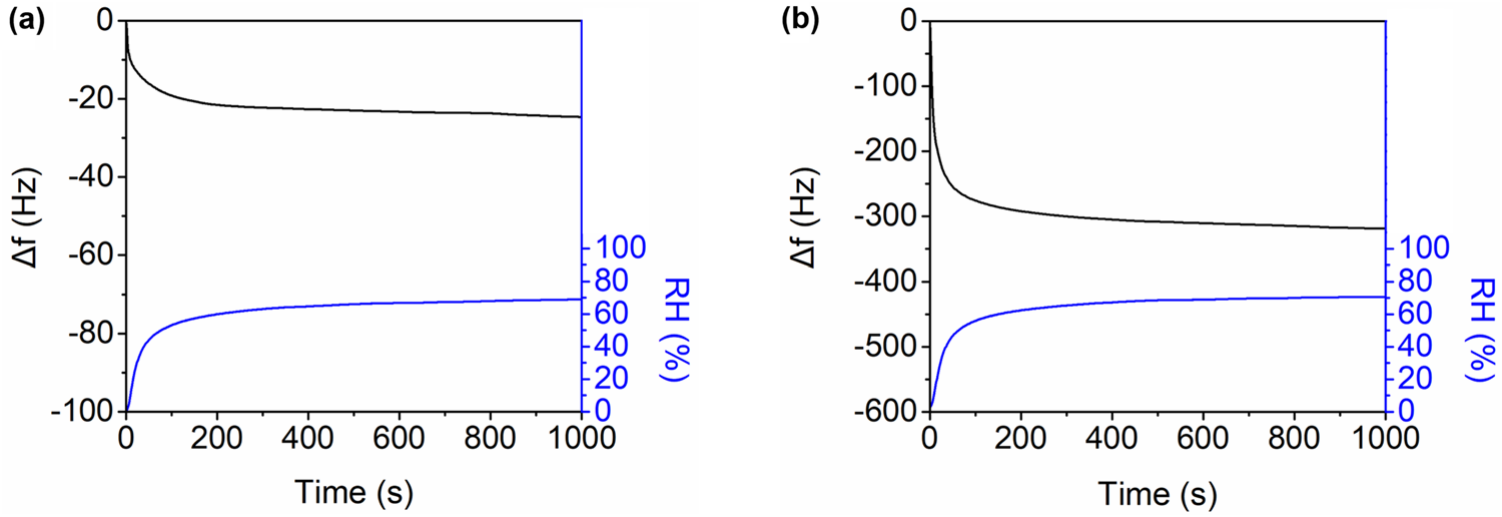
Time-dependent changes in the resonant frequency (black) of (**a**) a bare QC and (**b**) an SCP–QC during humidification as RH increased to 70%. Blue curves indicate changes in RH during humidification.

Figures 4a,b show the time-dependent changes in the resonant frequency of the bare QC (i.e., QC without an SCP coating) and SCP–QC, respectively, during humidification as the RH increased to 70%. With the initiation of humidification, the RH increased from 0 to 70%, causing a decrease in the resonant frequency. The maximum reduction in frequency for the bare QC was only 25 Hz, whereas a substantial decrease of 325 Hz was observed for the SCP–QC. The time-dependent changes in the resonant frequency were best fitted with the following second-order exponential function:

1 $$\Delta f\left({\text{t}}\right)={D}_{1}\left(1-{\text{exp}}\left(\frac{t}{{\tau }_{1}}\right)\right)+{D}_{2}\left(1-{\text{exp}}\left(\frac{t}{{\tau }_{2}}\right)\right)$$

where Δ*f*(*t*) is the resonant frequency shift at time *t*; *D*_*i*_ is the resonant frequency shift contributed by the *i*^th^ sorption component; and τ_*i*_ is time constant of the *i*^th^ sorption component^12–14^. The changes in the time-dependent resonant frequency of the bare QC and SCP–QC were best fit with Eq. (1) (coefficient of determination, *R*^2^ > 0.99, Supplementary Fig. S3). The best-fit values of *D1*, *D2*, *τ*_1_, and *τ*_2_ are listed in Table 1. Note that *τ*_2_ is one order of magnitude larger than *τ*_1_, suggesting that moisture adsorption occurred through two distinct processes. In addition, the short time constant *τ*_1_ increased twofold in the presence of the SCP coating, whereas the long time constant *τ*_2_ remained nearly constant regardless of the presence of the SCP coating. The fast process was attributed to the direct adsorption of water molecules on the surface, influenced by surface chemistry, whereas the slow process was attributed to the adsorption of water molecules on the adsorbed water (i.e., multilayer adsorption) and remained unaffected by the surface type^15–17^. A control analysis showed that the changes in the resonant frequency did not fit well with a first-order exponential function, confirming the presence of two distinct moisture adsorption processes (Supplementary Fig. S4).

**Table 1:** The best-fit values with Eq. (1) for changes in the resonant frequency of the bare QC and SCP–QC as RH varies from 0 to 70%.

QC	*D*_1_ (Hz)	τ_1_ (s)	*D*_2_ (Hz)	τ_2_ (s)
Bare QC	− 9.4	3.8	− 12.8	69.7
SCP–QC	− 200.4	7.2	− 96.2	64.2

**Figure 5:**
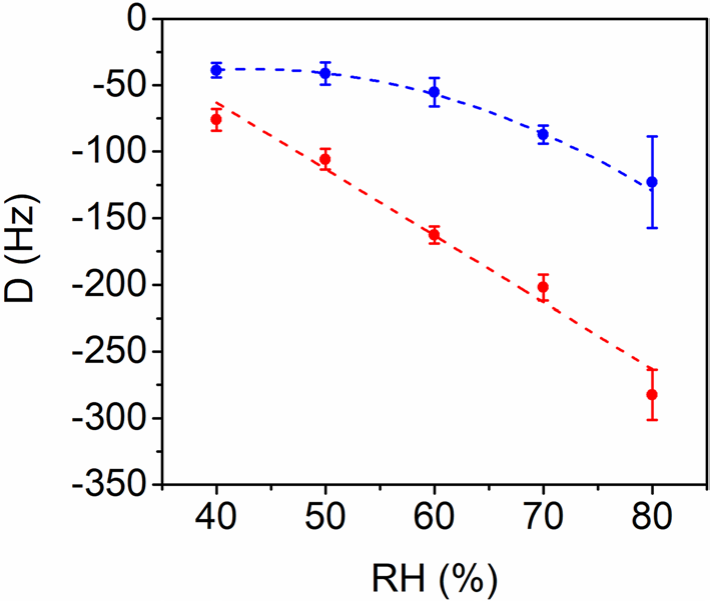
Variations in *D*_1_ (red) and *D*_2_ (blue) as a function of RH. *D*_1_ varied linearly with increasing RH, whereas *D*_2_ exhibited more significant variation at higher RH.

The time-dependent changes in the resonant frequency under different RH conditions (Fig. 3a) were fitted with Eq. (1) using *τ*_1_ (7.2) and *τ*_2_ (64.2) obtained at 70% RH. The results demonstrated a good fit (*R*^2^ > 0.98) with Eq. (1) under all RH conditions (Supplementary Fig. S5). Figure 5 shows the variations in *D*_1_ and *D*_2_ of SCP–QC as RH increases. The absolute values of both *D*_1_ and *D*_2_ increased as RH increased, indicating an increase in adsorption with higher RH conditions. Interestingly, *D*_1_ exhibited a linear variation with increasing RH, whereas *D*_2_ showed more significant changes at higher RH conditions, suggesting that multilayer adsorption became more prominent at higher RH conditions.

**Figure 6:**
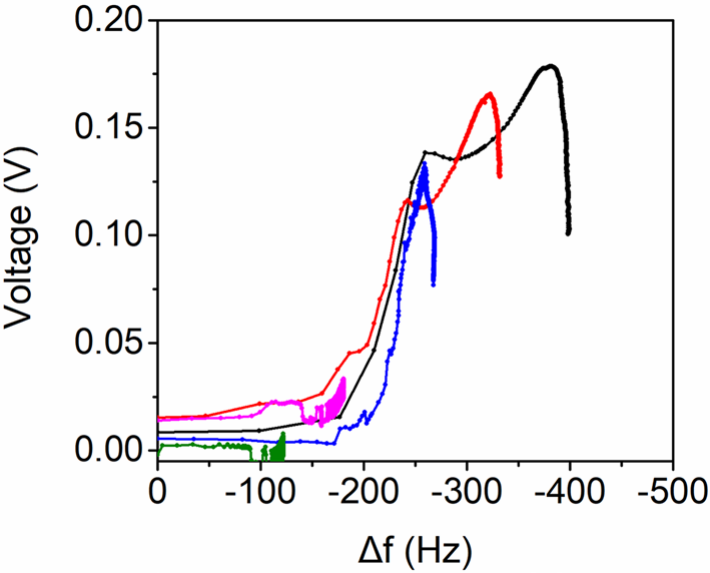
Changes in the open-circuit voltage as a function of resonant frequency when the SCP–QC was exposed to different maximum RH conditions: 80% (black), 70% (red), 60% (blue), 50% (pink), and 40% (green).

Figure 6 shows the changes in the open-circuit voltage as a function of the resonant frequency when the SCP–QC was exposed to different RH conditions: 40% RH (SQ40), 50% RH (SQ50), 60% RH (SQ60), 70% RH (SQ70), and 80% RH (SQ80). Three distinct regions were observed in the voltage outputs during moisture adsorption. In the early stage, the frequency change increased because of moisture adsorption, but the change in voltage output was negligible because the adsorbed water was insufficient to enable ion migration. In the middle stage, the changes in the frequency and voltage output increased until they reached their maximum values, indicating that ion migration was facilitated with increased water adsorption. Because the voltage output was generated only under sufficiently high RH conditions, SQ40 and SQ50 did not reach this region. In the late stage, the voltage outputs of SQ60, SQ70, and SQ80 decreased with frequency changes due to the formation of water channels between the electrodes. A control experiment confirmed that the electrical resistance of the SCP film decreased to a greater extent at higher RH (Supplementary Fig. S6). The decrease in the internal resistance, in turn, leads to diminished voltage generation.

## Conclusion

In this study, we investigated the real-time relationship between voltage generation and moisture adsorption by fabricating an MPG on a CIDE–LFE quartz resonator; to the best of our knowledge, this is the first time that such an attempt has been made. Voltage output and gravimetric measurements, when conducted separately, provided different insights. Voltage output measurements showed a rapid increase in voltage upon the initiation of humidification until it reached a maximum value and a subsequent decrease in the voltage. Gravimetric measurements, on the other hand, revealed two distinct water adsorption dynamics: water adsorption on the SCP surface and multilayer water adsorption. In contrast, the simultaneous measurements of voltage generation and the resonant frequency of SCP–QC revealed three distinct regions in the voltage outputs during moisture adsorption: a region of negligible voltage generation, a region with increasing voltage generation, and a region with decreasing voltage generation. These results highlight the utility of the developed method in elucidating the voltage-generation process of MPG and improving MPG performance through the identification of suitable materials.

### Supplementary Information

Supplementary Figures.

## Data Availability

All data required to reproduce the results can be obtained from the authors by contacting H.B. or S.J.
