# Supplementary material for: Moisture-induced power generator fabricated on a lateral field-excited quartz resonator
Source: Sci Rep. 2024 May 11;14:10817. doi: 10.1038/s41598-024-61669-0 (PMC11088684; doi:10.1038/s41598-024-61669-0)
Supplement: Supplementary file 1 — Supplementary Figures. [file 41598_2024_61669_MOESM1_ESM.pdf]

Supplementary Information

Moisture-Induced Power Generator Fabricated on a Lateral Field Excited Quartz Resonator

Hyerim Baek, Jihun Choi, and Sangmin Jeon*

Department of Chemical Engineering, Pohang University of Science and Technology (POSTECH), 77 Cheongam-Ro, Pohang, Gyeongbuk, Republic of Korea

* Author to whom correspondence should be addressed. E-mail: jeons@postech.ac.kr

Number of Pages: 7

Number of Figures: 6

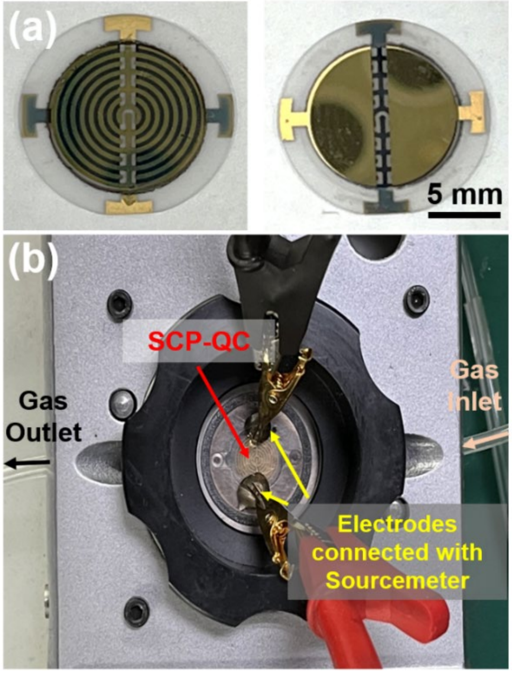

Figure S1. (a) Photo of the top surface (left) and bottom surface (right) of the fabricated SCP-QC. (b) Top view of humidity controlled flow cell. The SCP-QC was placed in the humidity controlled flow cell with IDEs of SCP-QC were positioned upward. Two LFE electrodes which located on the bottom surface of the SCP-QC were in contact with two pogo pins connected to the impedance analyzer (QCM Z500), and two IDEs which located on the top surface were in contact with two pogo pins connected to the sourcemeter.

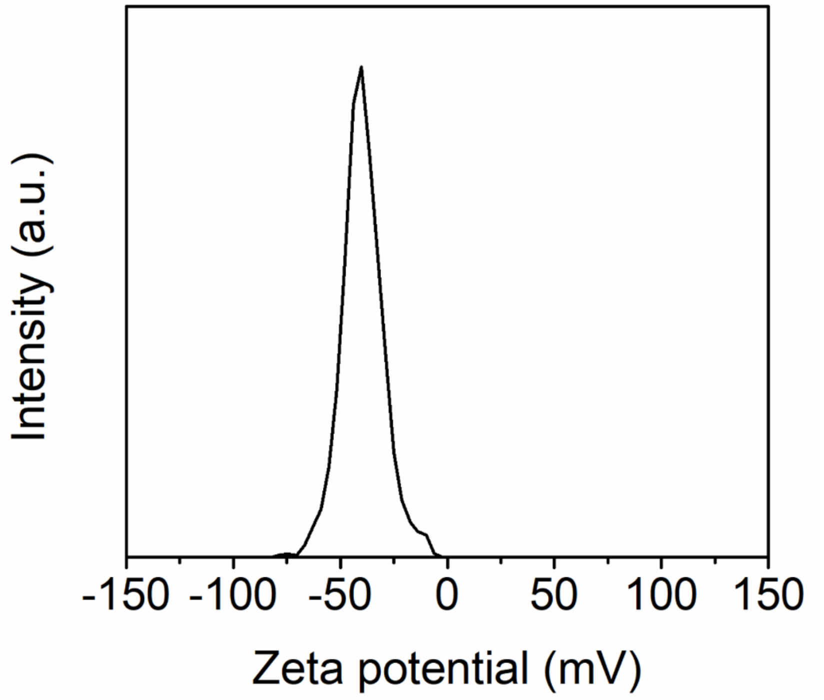

Figure S2. The zeta potential distribution of CB. The peak of the distribution has a value of -40.2 mV.

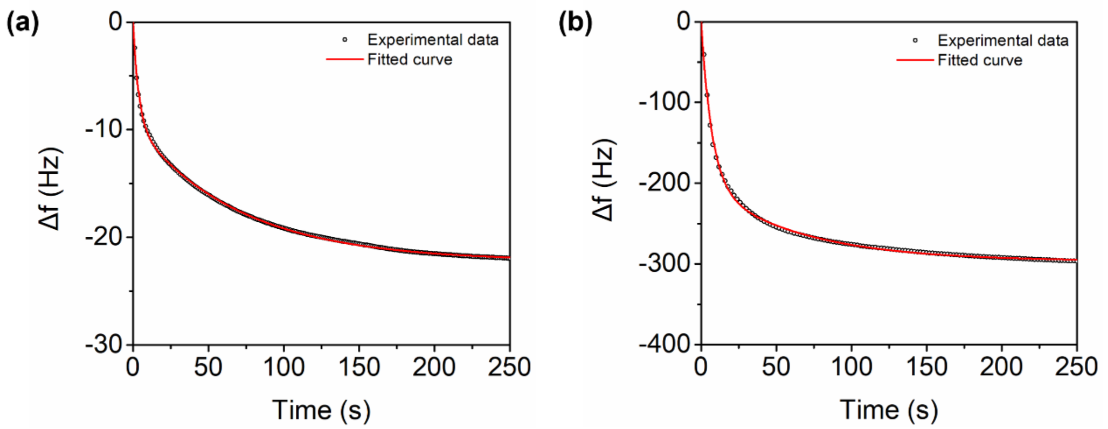

Figure S3. Time-dependent changes in the resonant frequency of (a) the bare QC and (b) the SCP-QC under 70% RH condition. The experimental data (black) were fitted with the second-order exponential function: $\Delta f(t) = D_1\ (1 - \exp\ (t\ /\ \tau_1)) + D_2\ (1 - \exp\ (t\ /\ \tau_2))$. The coefficient of determinations (R^2) were higher than 0.99 in both cases.

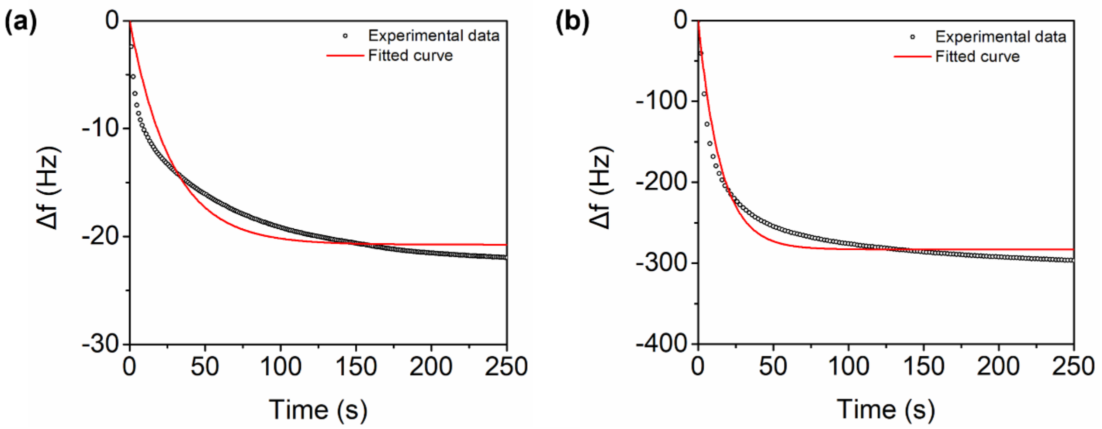

Figure S4. Time-dependent changes in the resonant frequency of (a) the bare QC and (b) the SCP-QC under 70% RH condition. The experimental data (black) were not well fitted with the first-order exponential function: $\Delta f(t) = D_1\ (1 - \exp\ (t\ /\ \tau_1))$.

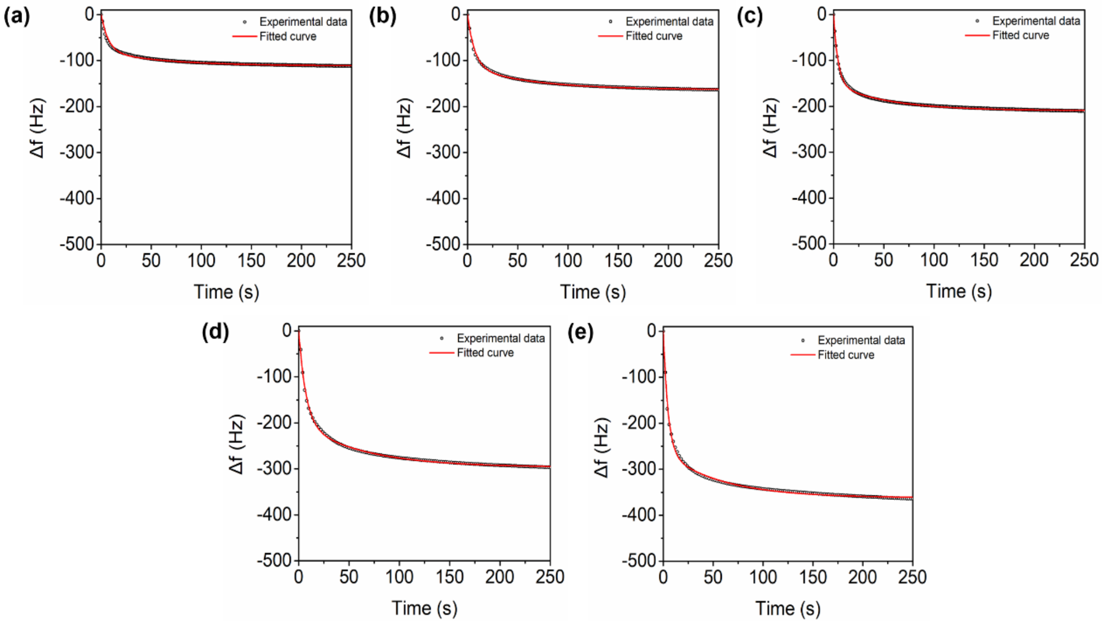

Figure S5. The time-dependent changes in the resonant frequency of SCP-QC were fitted to the second-order exponential function with time constants of τ_1 (7.2) and τ_2 (64.2) under (a) 40%, (b) 50%, (c) 60%, (d) 70%, and (e) 80% RH conditions. The coefficient of determinations (R^2) were higher than 0.98 in all cases.

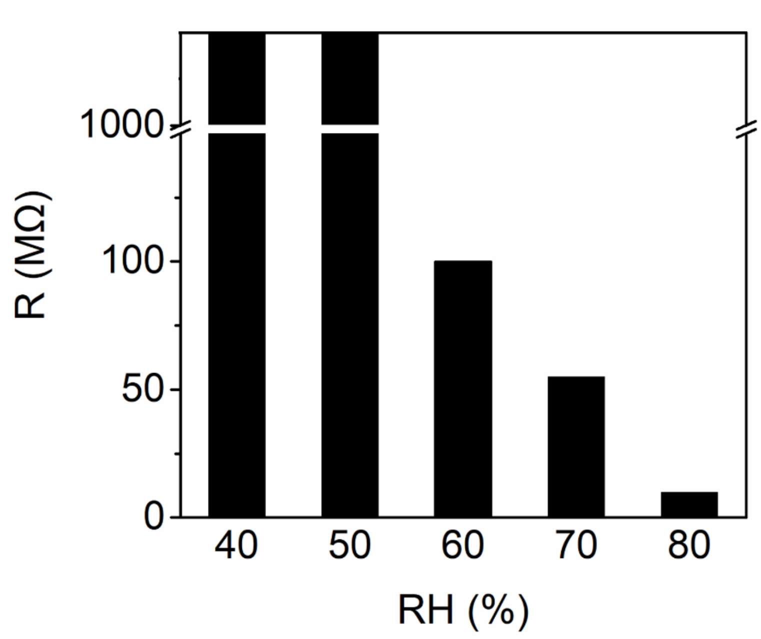

Figure S6. Variations in the electrical resistances between CIDEs of SCP-QC under various RH conditions. The electrical resistance decreased as RH increased: >1000 MΩ at 40% and 50%, 100 MΩ at 60%, 55 MΩ at 70%, and 10 MΩ at 80%.
